# Na[^18^F]F PET/CT quantification in spondyloarthritis: comparative evaluation of SUV normalization approaches

**DOI:** 10.1186/s13550-025-01337-0

**Published:** 2025-12-24

**Authors:** Wouter R. P. van der Heijden, Sam Groothuizen, Gerben J. C. Zwezerijnen, Robert C. Schuit, Robert Hemke, Ronald Boellaard, Conny J. van der Laken, Maqsood Yaqub

**Affiliations:** 1 Amsterdam UMC, Department of Clinical Immunology and Rheumatology, Meibergdreef 9, Amsterdam, Netherlands; 2 Amsterdam UMC, Department of Radiology and Nuclear Medicine, De Boelelaan 1117, Amsterdam, Netherlands

**Keywords:** [^18^F]Fluoride PET, Spondyloarthritis, SUV quantification, Kinetic modelling

## Abstract

**Background:**

Sodium [^18^F]Fluoride Positron Emission Tomography (Na[^18^F]F PET) is a promising imaging biomarker for evaluating bone metabolism in spondyloarthritis (SpA) and other bone affecting diseases. Accurate quantification of tracer uptake is essential for assessing disease activity and treatment response. This study aimed to determine optimal simplified metrics for Na[^18^F]F uptake and evaluate their performance compared to net influx rate (*K*_*i*_).

**Results:**

A prospective study included 54 SpA patients undergoing Na[^18^F]F PET/CT scans at baseline and after 12 weeks of therapy. Dynamic PET in combination with venous blood sampling was analyzed to derive kinetic parameters, including *K*_*i*_ in 43 scans that included pathological uptake in the dynamic field of view. Semi-quantitative standardized uptake values (SUVs) corrected body weight (BW), lean body mass (LBM), body surface area (BSA) and skeletal volume (SV) were compared and correlations between *K*_*i*_ and SUVs were assessed cross-sectionally and longitudinally. Based on analysis of the blood sample data, there was a significant difference between SUV corrected for BW between patients who weighted more and less than 85 kg (p < 0.01 at all sample moments). When LBM or SV was used, this difference disappeared (p > 0.05). There was a significant correlation between *K*_*i*_ and various SUV-metrics, with SUV_peak-LBM_ at 25–30 min yielding the highest correlation both cross-sectionally (R^2^ = 0.77, p < 0.01), and longitudinally (R^2^ = 0.54, p < 0.01).

**Conclusions:**

Na[^18^F]F uptake quantification of lesions in the axial skeleton of SpA patients can be performed cross-sectionally and longitudinally with simplified uptake measures, particularly SUV_peak_, normalized using LBM or SV. This offers a more reliable approach to evaluating disease activity and treatment responses compared to BW.

**Supplementary Information:**

The online version contains supplementary material available at 10.1186/s13550-025-01337-0.

## Introduction

The application of Sodium [^1^⁸F]Fluoride (Na[^1^⁸F]F) PET-CT has enhanced our ability to study bone metabolism in vivo, providing new insights into its role in inflammatory conditions such as spondyloarthritis (SpA) [1–6]. SpA encompasses a group of chronic inflammatory diseases, including axial SpA (axSpA) and psoriatic arthritis (PsA), which are characterized by both inflammatory and structural changes in bone and surrounding tissues [7]. A critical component of assessing disease activity and therapeutic response in SpA is the ability to quantify bone metabolism and remodeling at affected sites from early stages of the disease onwards and during treatment. While traditional imaging methods like MRI and X-ray focus on structural changes and inflammation, Na[^18^F]F PET-CT offers unique insights into bone metabolic activity and in particular molecular new bone formation as hallmark of SpA disease activity [3, 8, 9].

Na[^18^F]F PET indicates the uptake of fluoride ions into hydroxyapatite crystals, reflecting osteoblastic activity [10]. Given the small volumes of the lesions, accurate quantification may be challenging due to factors such as motion artifacts, partial volume effects (PVEs), and input function calibration. Dynamic PET imaging, which involves capturing time-activity curves (TACs) over the course of tracer uptake, provides detailed kinetic parameters such as the net influx rate (*K*_*i*_). This kinetic analysis requires plasma input functions derived from either blood sampling or image-derived methods. While highly accurate, dynamic imaging is time-intensive, requires technical expertise, and is less feasible in routine clinical practice.

To address these limitations, simplified approaches such as the standardized uptake value (SUV) have been proposed [11–15]. SUV is a static metric that normalizes tracer uptake for injected tracer activity and distribution volumes such as body weight (BW), lean body mass (LBM), or body surface area (BSA) [16, 17]. SUV is calculated from a single imaging time point, making it more practical for clinical applications. However, the accuracy of SUV depends on several factors, including the used normalization approach. Currently, in most studies that perform PET quantification, BW is used as the normalization factor. Studies in oncology have demonstrated that LBM-based normalization often reduces variability compared to BW normalization when there is low tracer uptake in the adipose tissue [11, 18–20]. Also, the use of skeletal volume (SV) as a normalization factor has been suggested as a more biologically relevant approach, given that Na[^18^F]F primarily localizes to bone [21, 22].

Although Na[^18^F]F PET-CT has been extensively studied in oncology and metabolic bone disorders, its application in inflammatory arthritis, particularly SpA, is still emerging. Studies evaluating the relationship between SUV, kinetic parameters, and clinical outcomes in SpA are limited [13]. Most published research has focused on small cohorts, and there is little consensus on the optimal methods for normalizing and interpreting static PET data.

This study aimed to address several key gaps in the field. First, we apply different normalization factors to the activity concentration in venous samples to illustrate the rationale for normalization. Secondly, we evaluate the feasibility of using SUV as a simplified measure of bone metabolic activity in SpA patients. Lastly, we assessed the value of SUV in the quantification of longitudinal changes in bone metabolism, caused by various biological disease modifying anti-rheumatic drugs (bDMARDs).

## Methods

A prospective study was conducted from October 2013 to June 2023, in which SpA (AxSpA and PsA) patients who were 18 years or older that had a clinical indication for biological therapy, were included. PsA patients were required to meet the CASPAR criteria and have at least one clinically active enthesitis based on SPARCC and/or MASES scoring system [23–25]. AxSpA patients had to fulfill the modified New York criteria and have a BASDAI score of ≥ 4 [26, 27]. Exclusion criteria included pregnancy, breastfeeding or the use of any experimental drug within the preceding three months. The use of conventional DMARDs or NSAIDs was permitted, provided the dosage remained stable for at least three months prior to inclusion and during the trial. PET-CT scans were acquired before initiation of bDMARD therapy and repeated after 12 weeks of treatment. The therapy targeted either tumor necrosis factor alfa (TNFα) (adalimumab or etanercept) or interleukin 17 A (secukinumab). The study protocol was approved by the Medical Ethics Review Committee of the VU University Medical Center, and all patients provided written informed consent.

### Image acquisition

PET-CT imaging was performed using either Ingenuity TF or Vereos systems (Philips Healthcare, Andover, MA, USA). An 18-gauge intravenous catheter was inserted into the antecubital vein of each arm; one for the administration of 100 MBq of Na[^18^F]F and the other for venous blood sampling. Following tracer injection, the catheter was flushed with 20 mL 0.9% NaCl. The residual activity in the syringe and tubing was measured to accurately determine the administered dose. Immediately after tracer injection, a 30-min dynamic PET acquisition of the chest (including the thoracic spine and the heart) was initiated. Venous blood samples (3 × 7 mL) were collected at 5, 15 and 25 min post- injection to calibrate input functions [28, 29]. Prior to the emission scan, a 30 mAs low-dose CT scan was performed for attenuation correction and anatomical localization. At 45 min post-injection, a whole-body static PET-CT scan was acquired from the skull base to mid-thigh, with an acquisition time of 3 min per field of view (FOV).

### Image reconstruction

PET data were normalized and corrected for attenuation, random coincidences, radioactive decay and scatter, following the EARL standards 1 [30]. All images were analyzed using a voxel size of 4 × 4 × 4 mm, as standardly generated by the reconstruction protocols in both camera’s. Dynamic scans were reconstructed into 25 frames with progressively increasing durations (1 × 10, 8 × 5, 5 × 20, 5 × 60, 3 × 150 and 3 × 300 s).

### Image analysis

Static PET-CT scans were independently reviewed by a board-certified musculoskeletal radiologist (RH) and a board-certified nuclear medicine specialist (GZ) both blinded for the clinical data. PET-positive lesions within the FOV of the dynamic scan were identified. In case of disagreement, adjudication was performed by a third expert reader (CL). Spherical volumes of interest (VOIs), with a volume of 1.2 cm^3^ were placed manually on PET-positive lesions on both the dynamic and static scan, with the hottest voxel of the lesion being the center of the VOI. In the dynamic scan, a spherical VOI with a 1.7 cm^3^ volume was drawn in the ascending aorta, to obtain an image-derived input function (IDIF). For each lesion in the dynamic FOV TACs were generated, to quantify the tracer uptake over time. Tracer uptake on the static scan was quantified using the maximum, peak and mean SUV (SUV_max_, SUV_peak_, SUV_mean_) inside the VOI.

### Skeletal volume

Since the static scans were acquired using a whole-body protocol, it was not possible to precisely determine the SV for each individual patient. To estimate the total SV, we calculated the combined volume of the spine, costae, pelvis, humeri, scapulae, clavicles, and sternum. This selection was based on prior analysis of a separate dataset of 61 rheumatoid arthritis patients with total-body low-dose CT scans, which was used to determine the average proportion of these bones relative to the total skeletal volume. Applying the TotalSegmentator algorithm to determine the volume of all bones in these cases yielded an average scaling factor of 1.80 (SD 0.07) [31, 32]. Since the volume of these bones, which are visible on whole-body PET/CT scans was found to reliably represent the overall SV, and because most scanning protocols involve whole-body rather than total-body imaging, it was decided to use the combined volume of these bones as a proxy for the total skeletal volume.

### Kinetic analysis

Plasma input functions were generated using the IDIF, which was calibrated with the venous blood samples. For every time point, the percentage difference between IDIF and the venous sample was calculated. The average of these calibration factors was then applied to adjust the entire IDIF. The adjusted IDIFs were visually inspected to confirm that the line matched the sample points, and preserved the shape of the sample data. Region-level TACs were subsequently analyzed using an irreversible two-tissue compartment (2t3k) plasma input model, using in-house built Matlab based non-linear fitting routines. This model yields kinetic parameters such as the net influx rate constant *K*_*i*_ (1/min), and is a common model for quantification of Na[^18^F]F uptake in bones [33].

### Venous sampling analysis

First, the variation of activity in the venous samples between patients was assessed. Blood SUVs were not used as an endpoint to evaluate the performance of normalization methods, but to demonstrate the necessity of appropriate normalization. SUVs were calculated for all (baseline and follow-up) samples, using BW, BSA, LBM (as calculated by the Janmahatsian method), and SV as normalization factor [34]. For each sampling timepoint and normalization method, the mean SUV and coefficient of variation (CoV) were assessed. The CoV is defined as the ratio of the standard deviation (SD) and the mean, with a low CoV representing low variation. Different factors, such as patient weight, sex, diagnosis and time point of the scan were compared to see if they influenced the SUV. Since weight is not a dichotomous variable, patients were classified into two groups, using a threshold of 85 kg. This threshold was chosen as an expected median weight based on previous SpA cohorts [1–3].

### Simplified metrics

The accuracies of several simplified static approaches were evaluated: SUV_peak_, SUV_mean_ and SUV_max_. These were calculated both for the static scan and for the last frame of the dynamic scan, which was acquired between 25 and 30 min after tracer injection. The SUVs were normalized using the four aforementioned approaches: BW, LBM, BSA and SV. Since it is unknown whether treatment with bDMARD alters tracer metabolism, the baseline and follow-up scans were analyzed separately and compared. To assess whether longitudinal comparisons of lesional uptake can be performed using simplified SUV-metrics, the ratio between *K*_*i*_ of lesions at follow-up and baseline were compared with the ratio between SUV at follow-up and baseline.

### Statistical analysis

Statistical analysis were performed using SPSS version 26.0 for Windows and Rstudio v4.3.2. Plots were generated using GraphPad Prism 10.2.0. Continuous variables are summarized using mean and SD or median and interquartile range (IQR) if the variables were not normally distributed. To allow comparison of variability across different SUV normalization methods, each set of SUVs was normalized by dividing by its own group mean, thereby yielding relative deviations. Levene’s test was then applied to these normalized distributions to assess whether the relative variances differed significantly between normalization methods. Activity concentrations in the blood samples between the groups were compared using independent sample T-tests. Test results with a p-value under 0.05 were considered significant. Simplified parameters were compared with *K*_*i*_ derived from the 2t3k model using the Pearson correlation coefficients. The same analysis was performed for the longitudinal changes. To test whether the correlation between *K*_*i*_ and the different SUV-metrics differed significantly, a Williams’ test for dependent, overlapping correlations was conducted. To assess potential treatment effects, linear regression analyses were performed with therapy type and SUV as independent variables, and *K*_*i*_ as dependent variable.

## Results

**Fig. 1:**
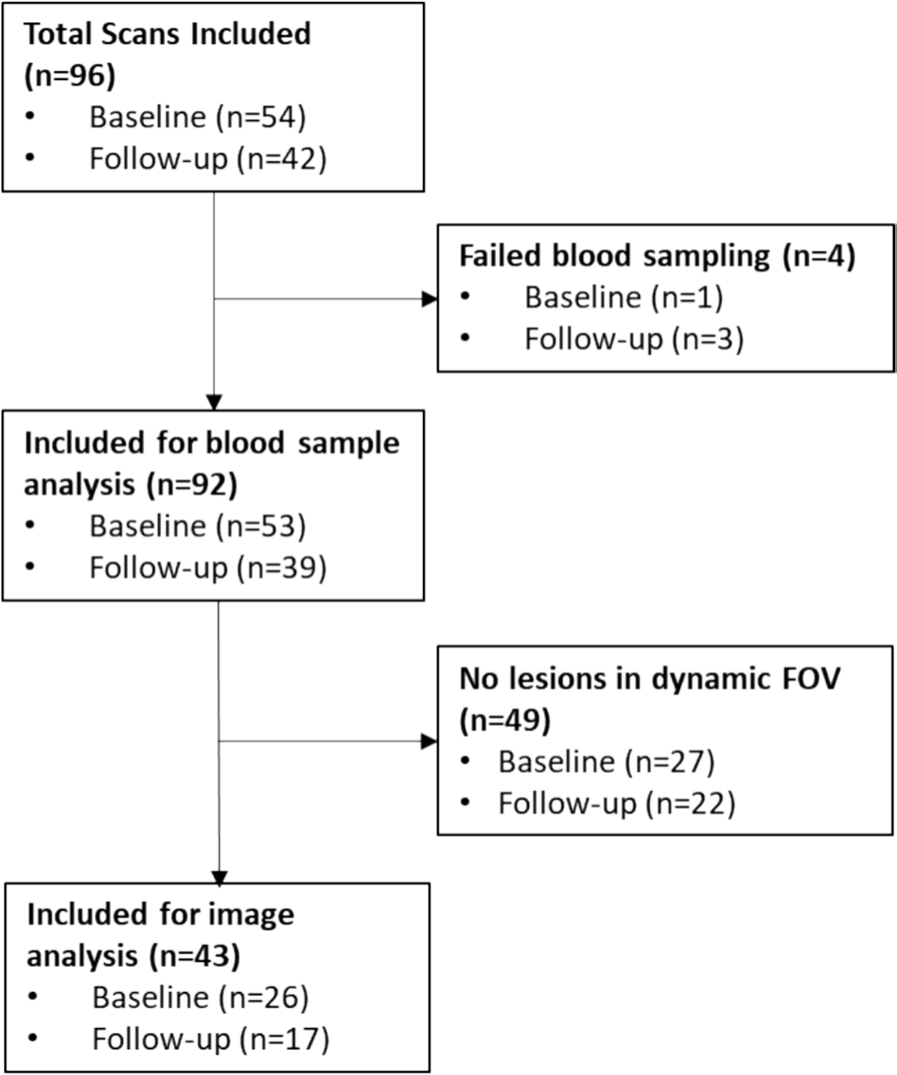
Flowchart of included patients and scans in the analysis of the venous samples and image analysis

Fifty-four SpA patients were included (31 with axSpA and 23 with PsA) (Table 1). Of these patients, 42 underwent follow-up scans leading to a total number of n = 96 scans. On average, 100.5 (± 5.0) and 101.5 (± 5.4) MBq Na[^18^F]F was injected at baseline and week 12, respectively. Due to failure of venous sampling in four cases, n = 92 scans were used in the sample analysis. N = 27 of the included patients did not have pathological uptake in the dynamic FOV, and were thus excluded from the image analysis, causing a total of n = 43 scans being used for the image analysis. These scans contained 16 pairs of baseline and follow-up scans, 10 baseline-only scans, and 1 follow-up only scan (in this patient the venous sampling failed at baseline) (Fig. 1). Slightly more patients started IL-17A therapy (n = 29, 55%) compared to anti-TNF therapy (n = 24, 45%). One patient did not start any bDMARD, despite being scheduled for anti-TNF. No follow-up scan was acquired in this patient. The patient characteristics of the groups that were used for each separate analysis can be found in Supplement 1. There were no substantial differences in patient weight between baseline and follow-up (median change 0.0 kg [IQR: –0.4 to 0.9 kg]), and no patient exhibited a weight change greater than 10 kg.

**Table 1:** Characteristics of patients included in image analysis

Characteristic	Patients included in image analysis (n = 27)
Diagnosis, no. (%) AxSpA PsA	19 (70%) 8 (30%)
Females, no. (%)	13 (48%)
Age, years	49.3 (13.5)
Height (cm)	172.9 (6.9)
Weight (kg)	86.2 (20.1)
Disease duration since diagnosis, years	5.9 [1.2–17.9]
CRP, mg/L	4.0 [2.4–11.5]
Therapy, no. (%) Anti-TNF Secukinumab	10 (38%) 16 (62%)

### Venous sampling

**Fig. 2:**
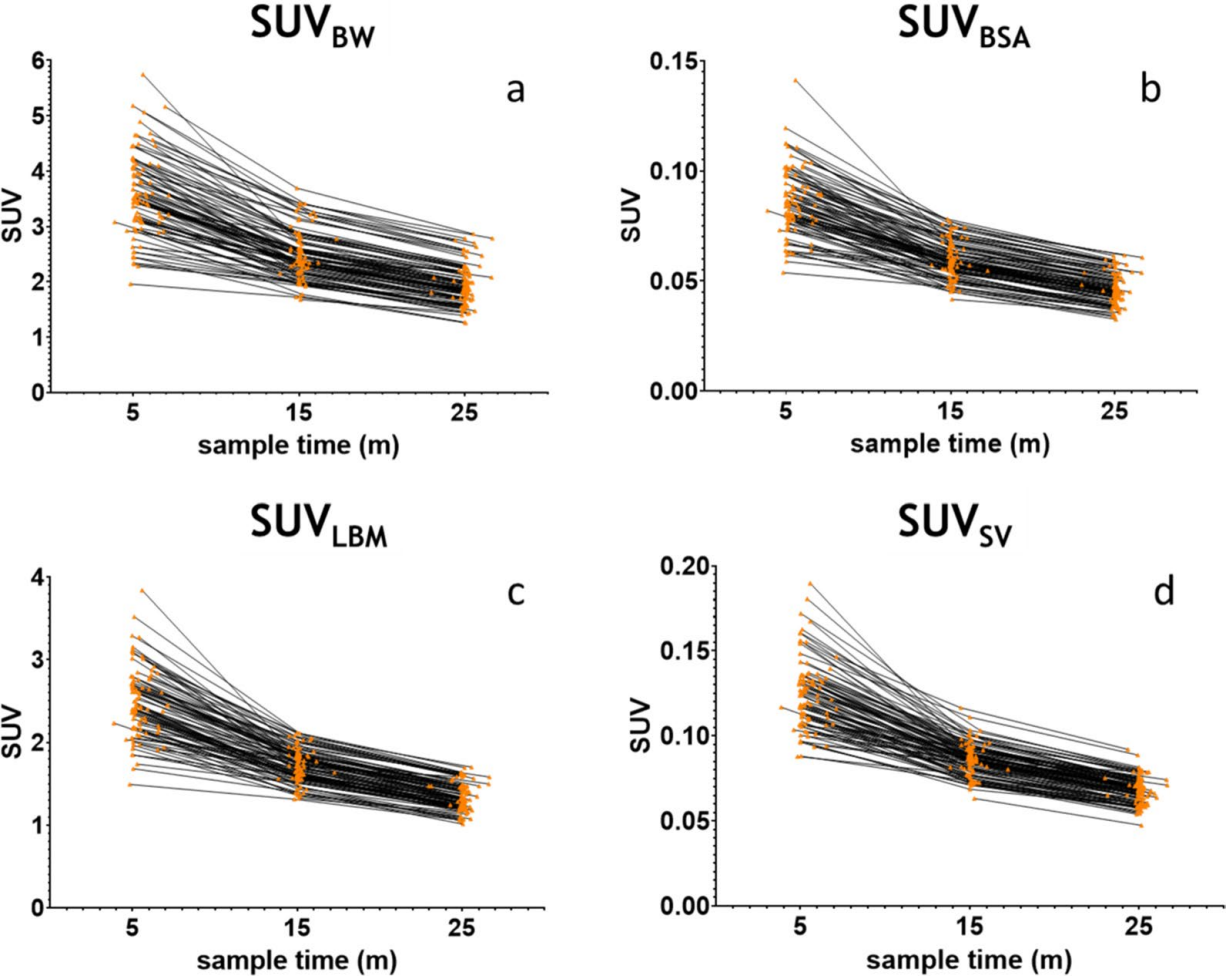
Time activity curves of the venous samples, as normalized for different factors. **a**: Body weight, **b**: Body surface area, **c**: Lean body mass, **d**: Skeletal volume

The SUV_bw_ in the venous blood samples showed the highest CoV for all sample moments, while SUV_LBM_ gave the lowest CoVs, which were very similar to SUV_BSA_ and SUV_SV_ (Table 2, Fig. 2). The observed differences in variability were statistically significant, as assessed using Levene’s test for equality of variances (p < 0.001). Calibration factors between the IDIF and samples showed no systematic trend across time points and no outliers were identified.

**Table 2:** Mean SUV, calculated with different normalization factors, in venous samples 5, 15 and 25 min after tracer injection, including coefficient of variation (CoV)

	Sample 1		Sample 2		Sample 3	
	mean	CoV	mean	Cov	mean	CoV
SUV_BW_	3.52	22%	2.47	19%	1.94	20%
SUV_BSA_ (× 10^2^)	8.54	19%	5.94	15%	4.66	17%
SUV_LBM_	2.44	19%	1.71	14%	1.34	14%
SUV_SV_ (× 10^2^)	12.29	20%	8.63	15%	6.76	14%

**Fig. 3:**
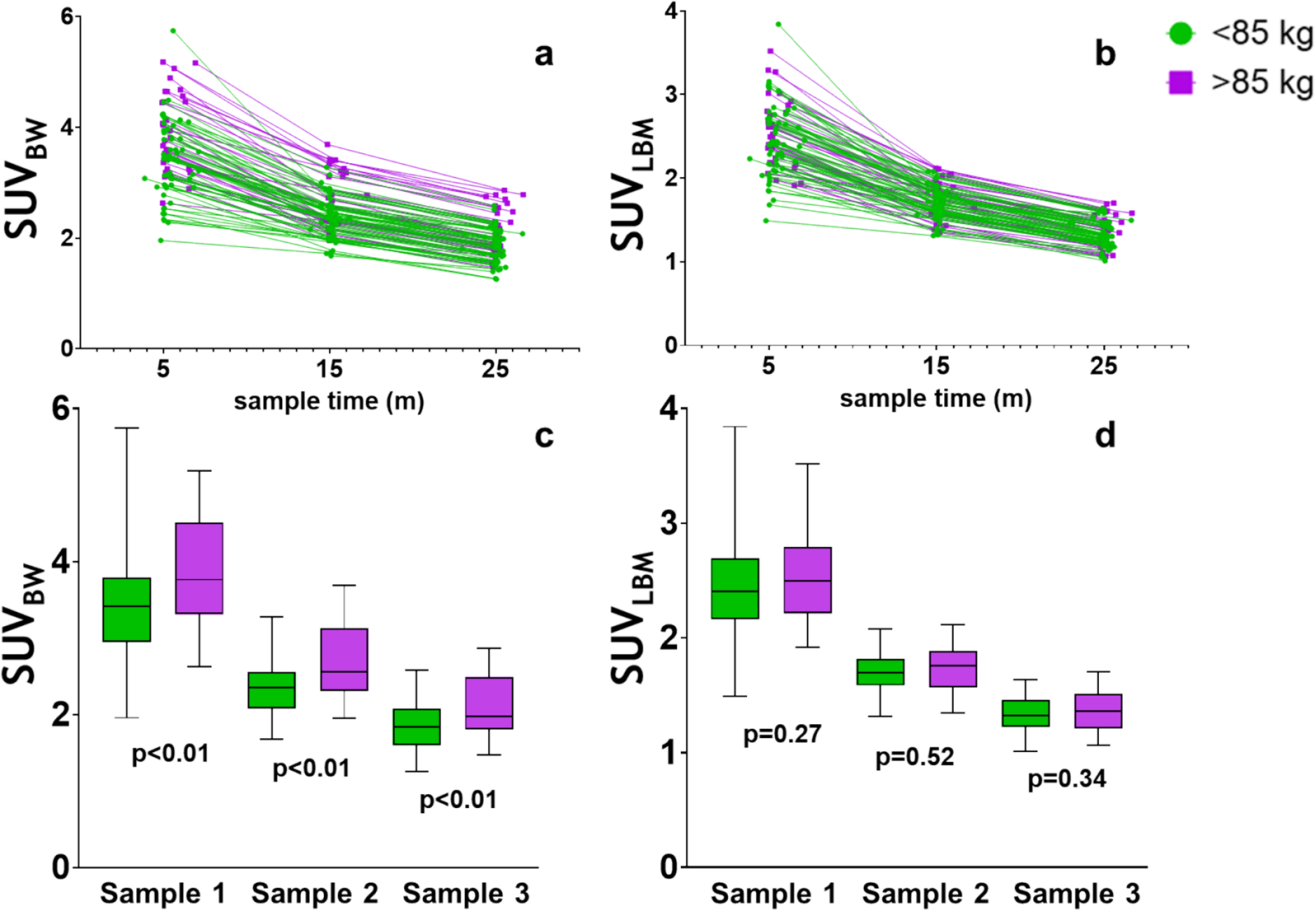
Time activity curves and boxplots of the venous samples normalized for body weight and lean body mass, with patients classified in groups based on weight: < 85 kg (green), and >85 kg (purple). **a**,**c**: Body weight, **b**,**d**: Lean body mass

When the tracer concentrations in the venous samples were corrected for injected activity and BW, a significant difference between the > 85 kg and < 85 kg group was found in the independent sample T-test (p < 0.01 at all sample moments). When LBM, BSA or SV were used, this difference disappeared (p > 0.05) (Fig. 3, Supplement 2). Furthermore, there were no significant differences in the blood tracer activity between timepoints, diagnosis (axSpA or PsA) and sex (all p > 0.05).

### Image analysis

**Fig. 4:**
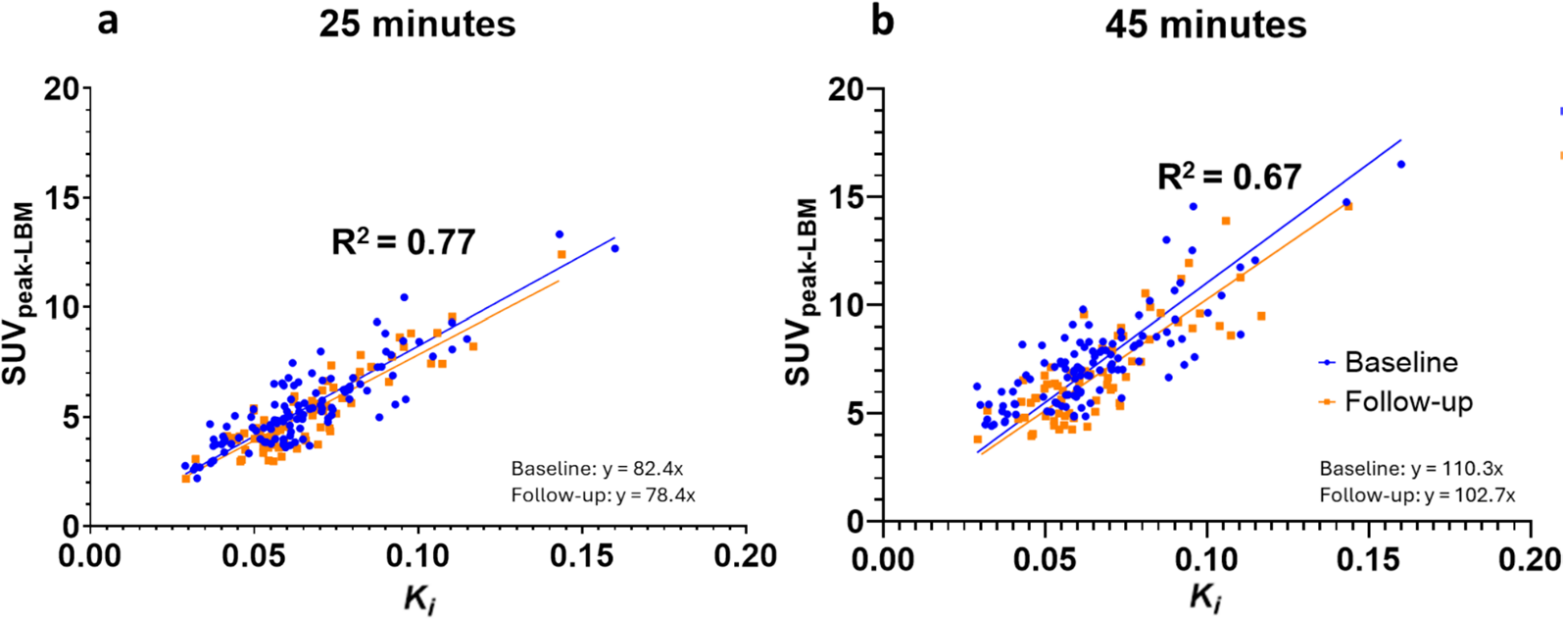
Scatter plot and linear regression line of the influx rate (K_i_) and SUV_peak_ normalized for lean body mass between 25 to 30 min after tracer injection (**a**) and of the influx rate (K_i_) and SUV_peak_ on the static scan 45 min after tracer injection (**b**) of all lesions at baseline (blue) and follow-up (orange)

In the n = 27 patients that had pathological uptake in the dynamic FOV, there was a significant correlation between *K*_*i*_ and all SUV-metrics, with SUV_peak_ at 25 to 30 min after tracer injection yielding the highest correlations (LBM: R^2^ = 0.77 [0.71–0.82], SV: R^2^ = 0.78 [0.72–0.83], BSA: R^2^ = 0.78 [0.72–0.83]) (Fig. 4a). When performing the Williams’ test there were no significant differences between these 3 normalization methods, but they all performed better compared to BW (R^2^ = 0.71 [0.63–0.77], p < 0.01). The slope of the regression curve, representing the correlation between SUV at the y-axis and *K*_*i*_ at the x-axis did not significantly change for SUV_peak-LBM_ between baseline (82.4, 95% confidence interval: 79.8–84.9) and follow-up (78.4 [75.6–81.1]).

When the *K*_*i*_’s were compared to the static scan, the SUV_peak-SV_ was found to be the most appropriate metric (R^2^ = 0.69 [0.60–0.76]), along with the SUV_peak-LBM_ (R^2^ = 0.67 [0.58–0.74]) and SUV_peak-BSA_ (R^2^ = 0.61 [0.52–0.69]) (Fig. 4b). All normalization methods performed significantly better than BW (R^2^ = 0.52 [0.41–0.61], p < 0.01), in the Williams’ test. In all normalization methods and times after injection SUV_peak_ and SUV_mean_ (R^2^ = 0.65 [0.56–0.72]) performed significantly better than SUV_max_ (R^2^ = 0.59 [0.49–0.67], p < 0.01). Bland–Altman plots are shown in the supplementary material (Supplement 5,6).

### Longitudinal analysis

**Fig. 5:**
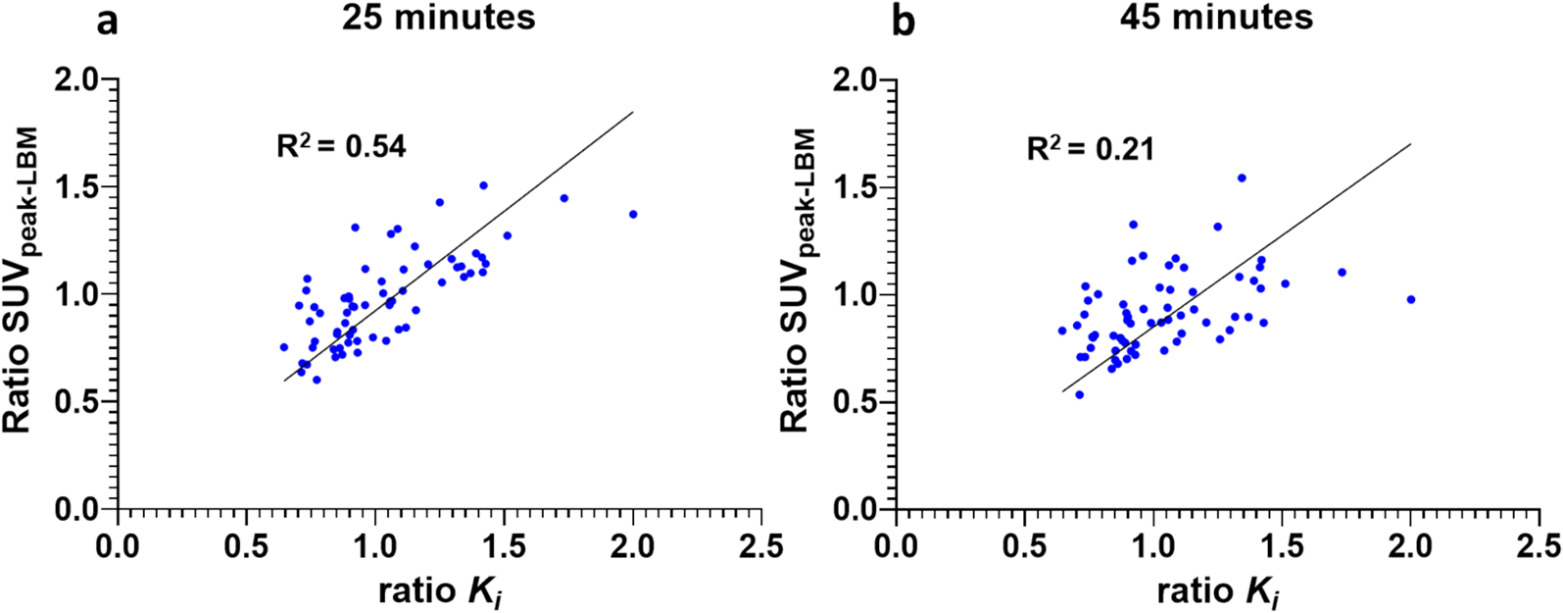
Scatter plot of the longitudinal ratio of K_i_ and SUV of lesions between baseline and follow-up at 25–30 min after tracer injection (**a**), and 45 min after tracer injection (**b**)

In the longitudinal analysis, changes in tracer uptake between baseline and follow-up scans were assessed. SUV_LBM_ at 25–30 min post-injection showed the strongest correlation with changes in *K*_*i*_ values (R^2^ = 0.54 [0.37–0.69])(Fig. 5). All other SUV normalization methods, including SV, BSA and BW demonstrated comparable correlations. Since the normalization factors were used at both baseline and follow-up and remained stable over time, this limited influence of the normalization factor was as expected. SUV_LBM_ after 45 min post-injection showed a low correlation (R^2^ = 0.21 [0.06–0.40]), which was similar to different normalization methods. In the regression analyses with treatment as a binary covariate, no statistically significant effect were observed between patients receiving the different therapies. (Supplement 3,4). Bland–Altman plots of the ratios were added to the supplementary material (Supplement 7,8).

## Discussion

This study aimed to identify reliable methods for simplifying the quantification of Na[^18^F]F uptake in SpA patients. Among the normalization approaches evaluated, LBM and SV emerged as the most suitable SUV normalization factors because of their ability to minimize variability in SUV measurements, particularly when compared to BW, which is currently the most prevalent approach for normalization. The LBM-based normalization demonstrated ease of use, as patient weight and height can be easily measured in clinical settings. In contrast, SV normalization requires additional computational steps, though it remains a viable alternative when LBM cannot be determined due to missing or unobtainable height and weight measurements. All our patients had a diagnosis of SpA which could have influenced tracer kinetics. However, it seems plausible that BW should also not be used in the quantification of Na[^18^F]F PET in other skeletal diseases, given the low uptake in adipose tissue that has been reported in previous research [18, 20]. This would open new opportunities for more accurate quantification and comparison of Na[^18^F]F PET, possibly leading to new insights that can improve patient management.

We found comparable results between SUV_peak_ and SUV_mean_, both outperforming SUV_max_. Both metrics can be used in this application, with a slight practical advantage for SUV_peak_ which is less reliant on the exact method of lesion segmentation and thus seems to be the preferable quantification metric. We did not observe significant differences in correlations between SUV and *K*_*i*_ between patients treated with anti-TNF or IL-17A inhibition. However, the number of patients in each subgroup, particularly in the anti-TNF group, was relatively small. Therefore, the absence of significant differences should be interpreted with caution, as the study was not powered to detect treatment-specific effects.

The higher correlation observed between *K*_*i*_ and SUVs calculated within the 25–30 min post-injection interval, compared to the static scan which was acquired 45 min after tracer injection, was most probably caused by VOI placement effects and patient motion. Since the SUVs at 25 min and the *K*_*i*_ values were calculated using the same VOIs, sampling differences likely had minimal influence on the correlation of the ratios at 25 min. In contrast, the correlation between *K*_*i*_ ratios and SUV at 45 min was based on four different VOIs, as they were defined separately on the whole-body scan, compared to only two for SUV at 25 min, which may have introduced additional variability and reduced the performance of SUV at 45 min. Even though the VOIs were placed around the voxel with the highest uptake, differences in voxel sampling could have caused variation in the quantification. Given the small size of the VOIs, these positional discrepancies may explain the reduced correlation observed between *K*_*i*_ and SUV metrics derived from static scans [35].

Another important factor that may have contributed to the lower correlations at 45 min is the influence of PVEs during the acquisition. PVEs occur when the spatial resolution of the PET system is insufficient to fully capture the true activity concentration in small lesions, leading to spill-out of signal into adjacent tissue. Both PVE and motion-induced misregistration could therefore have contributed to the observed reduction in correlation between SUV metrics and *K*_*i*_ at 45 min. The effect of patient motion on the IDIF was limited, since VOIs were checked and altered if necessary for the individual frames of the dynamic scans. However, PVEs may have affected the IDIF. While the magnitude of this effect remains uncertain, existing literature supports the notion that later scan times improve the performance of this tracer, possibly explaining that differences in voxel sampling account for much of the difference in correlation in this study [11, 13, 29, 36, 37]. It is a limitation of this study that we were not able to quantify this effect. Future studies should focus on a longer dynamic scanning protocol to reduce the noise introduced by VOI displacement. Besides SUV-based approaches, Patlak analysis should be considered as an intermediate solution between full kinetic modeling and static SUV measurements. Patlak analysis, which offers a less computationally demanding alternative for voxel-wise quantification and could provide more accurate quantification than SUV. The use of shortened Patlak protocols, such as recently studied for FDG-PET, may allow for broader clinical implementation [38]. These approaches are of high interest and should be explored in future studies.

The use of venous instead of arterial blood samples for Na[^1^⁸F]F kinetic modelling has been shown to provide accurate input function calibration when appropriate correction is applied, due to the rapid equilibration of fluoride between arterial and venous compartments after the first few minutes post-injection. Several studies have demonstrated strong agreement between venous and arterial activity concentrations for [^1^⁸F]fluoride PET beyond 5 min, indicating that venous sampling is a valid alternative for non-invasive studies [28, 29, 36]. We therefore adopted a venous sampling protocol as a less invasive and more patient-friendly method.

Another important limitation of this study is that only lesions within the dynamic FOV could be included in the analysis. As a result, the evaluation was restricted to lesions in the thoracic spine, and the kinetic behavior of lesions in the sacroiliac joints, as well as the lumbar and cervical spine, could not be assessed. Nevertheless, the thoracic spine contains all major spinal joint types, including facet joints, costovertebral joints, and costotransverse joints, making it a representative region for spinal assessment. Additionally, lesion selection was based on visual assessment, a subjective process that may have introduced variability and influenced the analysis outcomes.

## Conclusion

In a clinical setting, static imaging protocols are generally preferred over full dynamic acquisitions, as they require shorter scan times, are less burdensome for patients, and are more compatible with routine PET workflows. Our results support the use of LBM-based SUV_peak_ after 45 min over BW-based SUV for assessing bone metabolism in SpA and for therapeutic monitoring. This approach offers practical advantages, including its accessibility and reproducibility, while also demonstrating superior accuracy compared to BW normalization.

## Supplementary Information

Additional file 1.

## Data Availability

The datasets used and/or analysed during the current study are available from the corresponding author on reasonable request.
