# Supplementary material for: Na[18F]F PET/CT quantification in spondyloarthritis: comparative evaluation of SUV normalization approaches
Source: EJNMMI Res. 2025 Dec 24;15:143. doi: 10.1186/s13550-025-01337-0 (PMC12738459; doi:10.1186/s13550-025-01337-0)
Supplement: Supplementary file 1 — Additional file 1. [file 13550_2025_1337_MOESM1_ESM.docx]

Supplementary Files

| **Characteristic** | **All patients (n=54)** | **FU scan performed (n=17)** |
| --- | --- | --- |
| Diagnosis, no. (%)  AxSpA  PsA | 31 (57%)  23 (43%) | 9 (53%)  8 (47%) |
| Females, no. (%) | 30 (56%) | 9 (53%) |
| Age, years | 46.9 (12.4) | 50.2 (14.2) |
| Height (cm) | 173.6 (7.1) | 173.0 (7.9) |
| Weight (kg) | 83.2 (16.2) | 81.4 (14.4) |
| Disease duration since diagnosis, years | 5.5 [1.0 - 12.3] | 8.0 [2.0-18.7] |
| CRP, mg/L | 4.0 [2.4 – 10.8] | 8.0 [2.2-15.0] |
| Therapy, no. (%)  Anti-TNF  Secukinumab | 24 (45%)  29 (55%) | 5 (29%)  12 (71%) |

***Sup 1.*** *Characteristics of the patients included for the cross-sectional image analysis and the patients included for the longitudinal analysis*


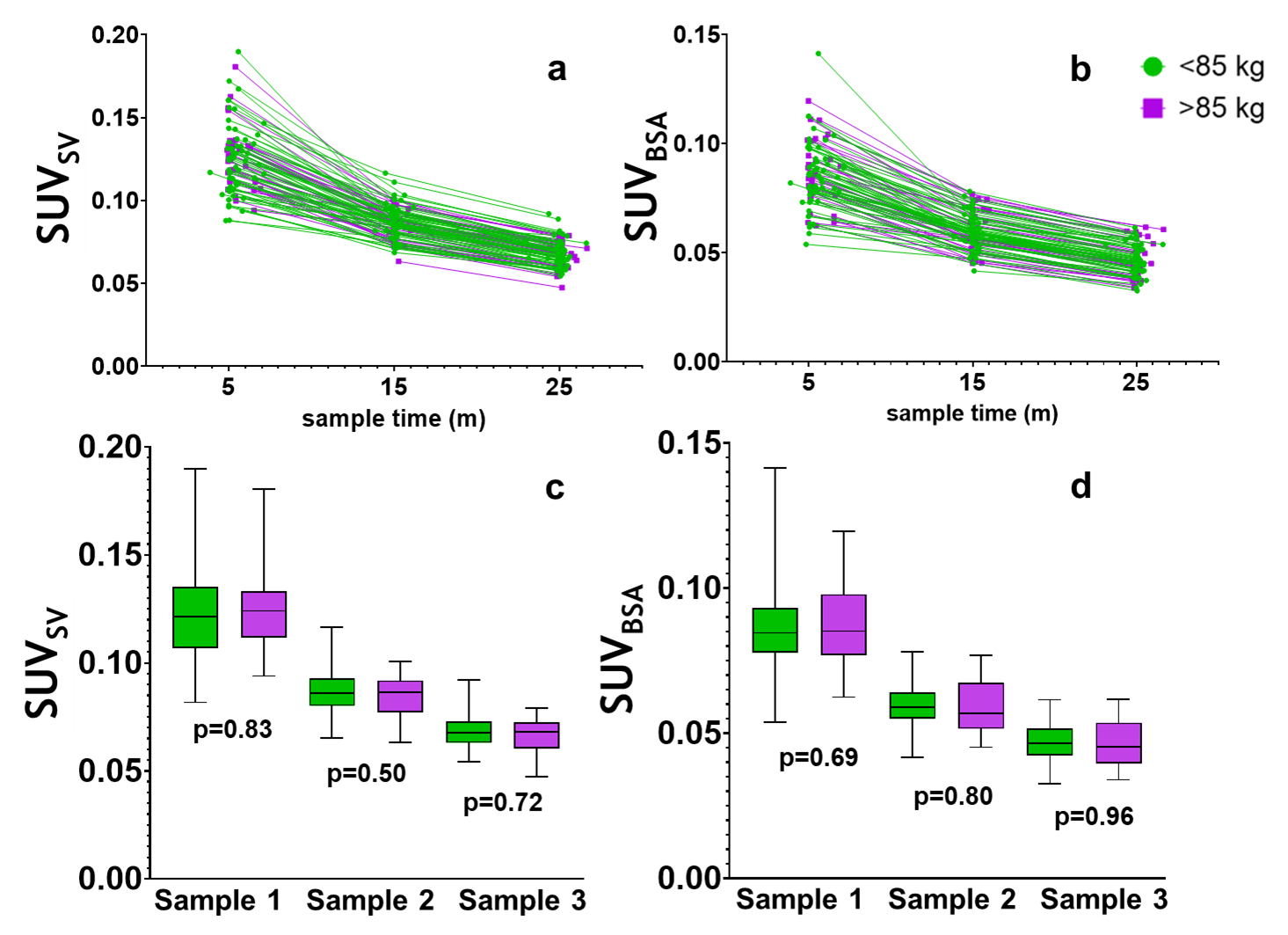


***Sup 2.*** *Time activity curves and boxplots of the venous samples normalized for skeletal volume and body surface area, with patients classified in groups based on weight: <85 kg (green), and >85kg (purple). a,c: skeletal volume, b,d: body surface area*

*
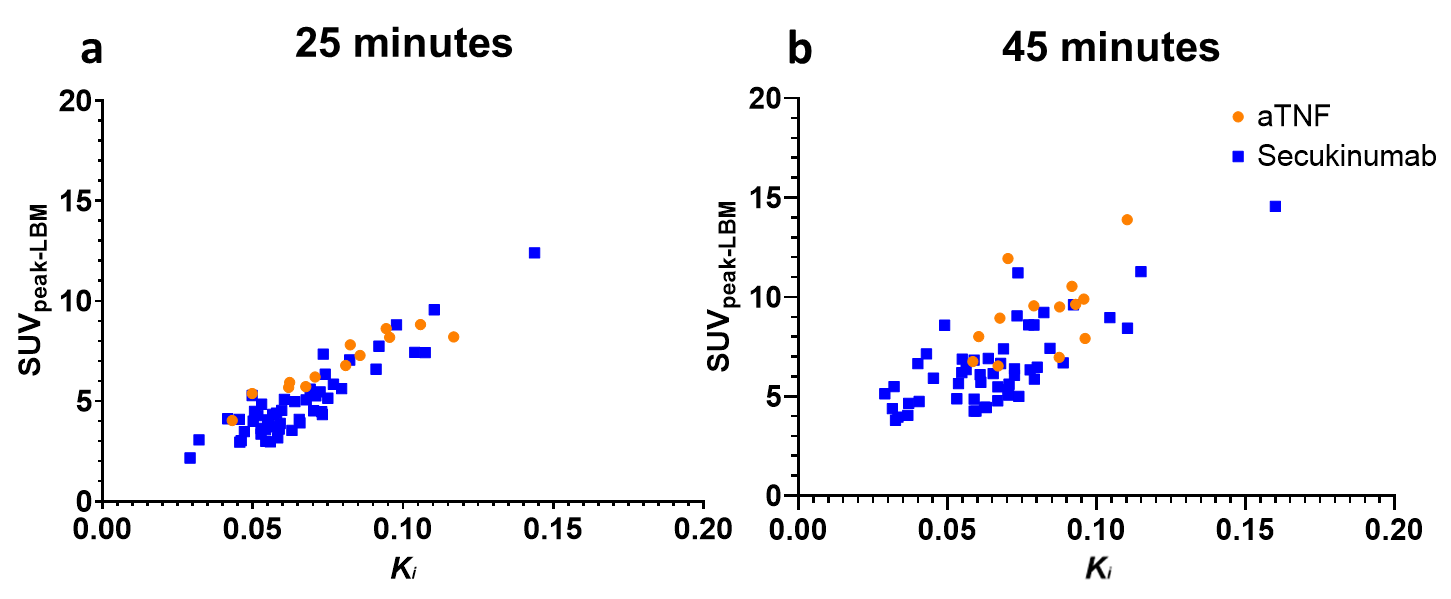
*

***Sup 3****. Scatter plots of the influx rate (Ki) and SUV_peak_ normalized for lean body mass in the lesions of the follow-up scans between 25 to 30 minutes after tracer injection (a) and of the influx rate (Ki) and SUV_peak_ on the static scan 45 minutes after tracer injection (b) of patients who started secukinumab treatment (blue) and anti-TNF treatment (orange).*

*
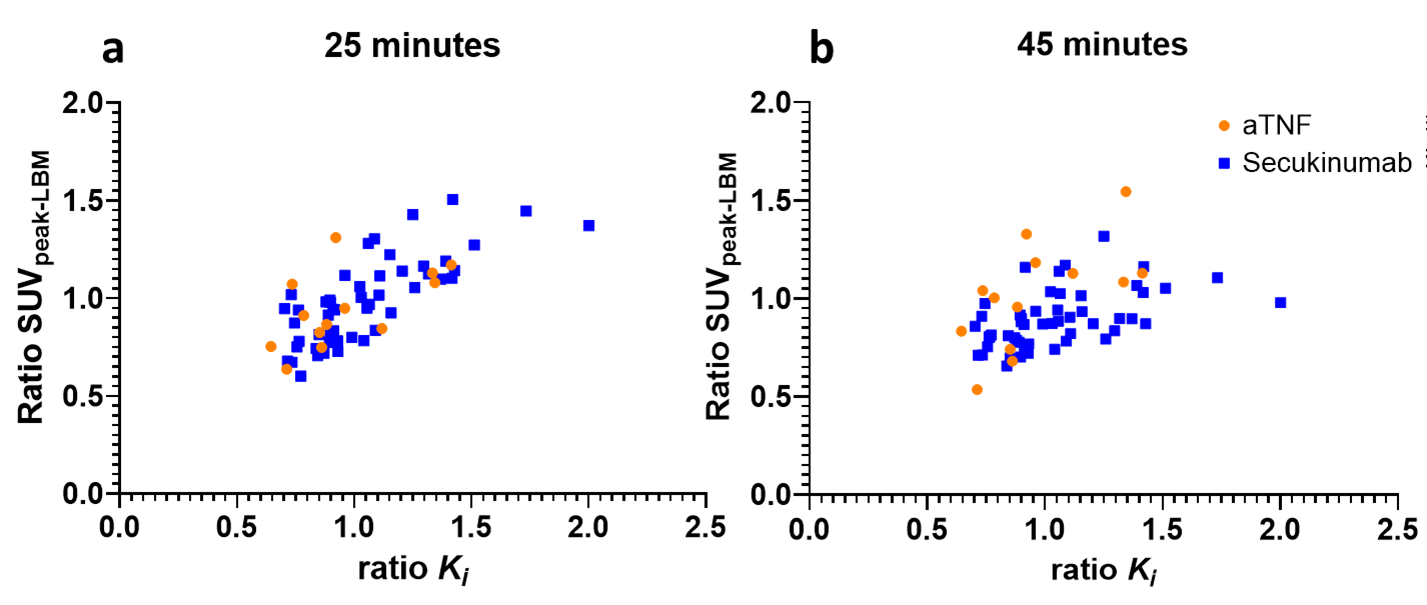
*

***Sup 4****. Scatter plots of longitudinal ratio of K_i_ and SUV of lesions between baseline and follow-up at 25-30 minutes after tracer injection (a), and 45 minutes after tracer injection (b) of patients who started secukinumab treatment (blue) and anti-TNF treatment (orange).*

*.*

*
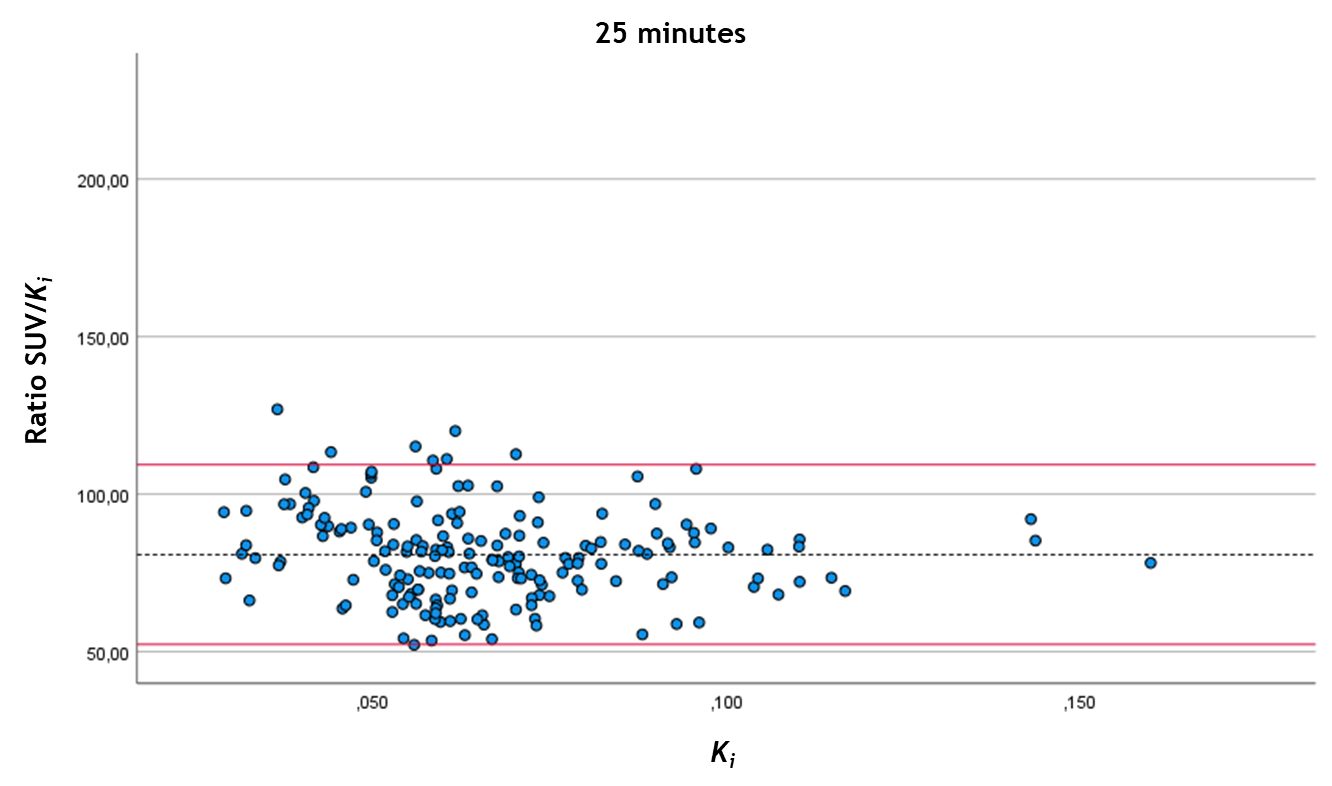
*

***Sup 5.*** *Bland-Altman plot of the ratio between SUV_peak-LBM_ and K_i_ at 25 minutes after tracer injection for each K_i_. The slightly higher ratio’s corresponding to lower K_i_’s may be explained by the blood volume fraction in the VOI, that does influence the SUV, but not the K_i._*

*
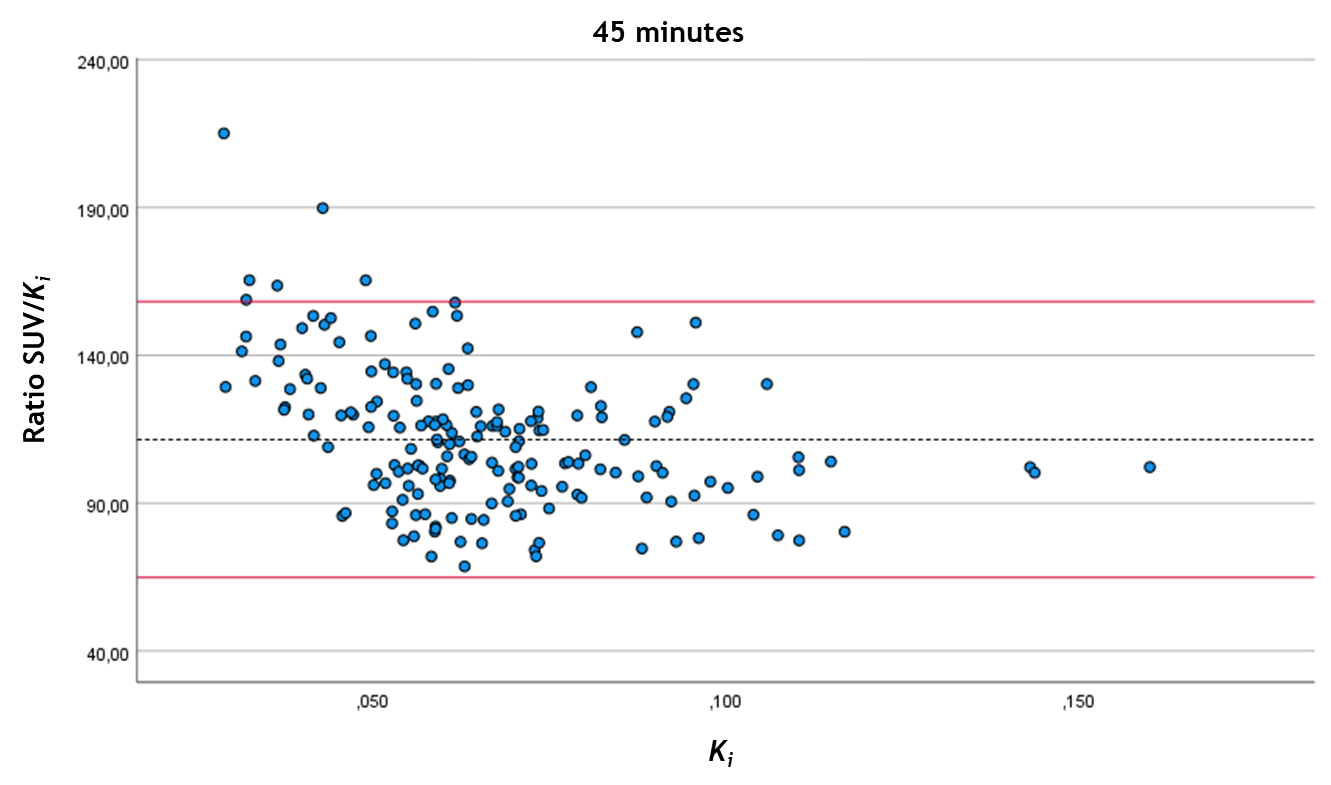
*

***Sup 6.*** *Bland-Altman plot of the ratio between SUV_peak-LBM_ and K_i_ at 45 minutes after tracer injection for each K_i_. The slightly higher ratio’s corresponding to lower K_i_’s may be explained by the blood volume fraction in the VOI, that does influence the SUV, but not the K_i._*

*
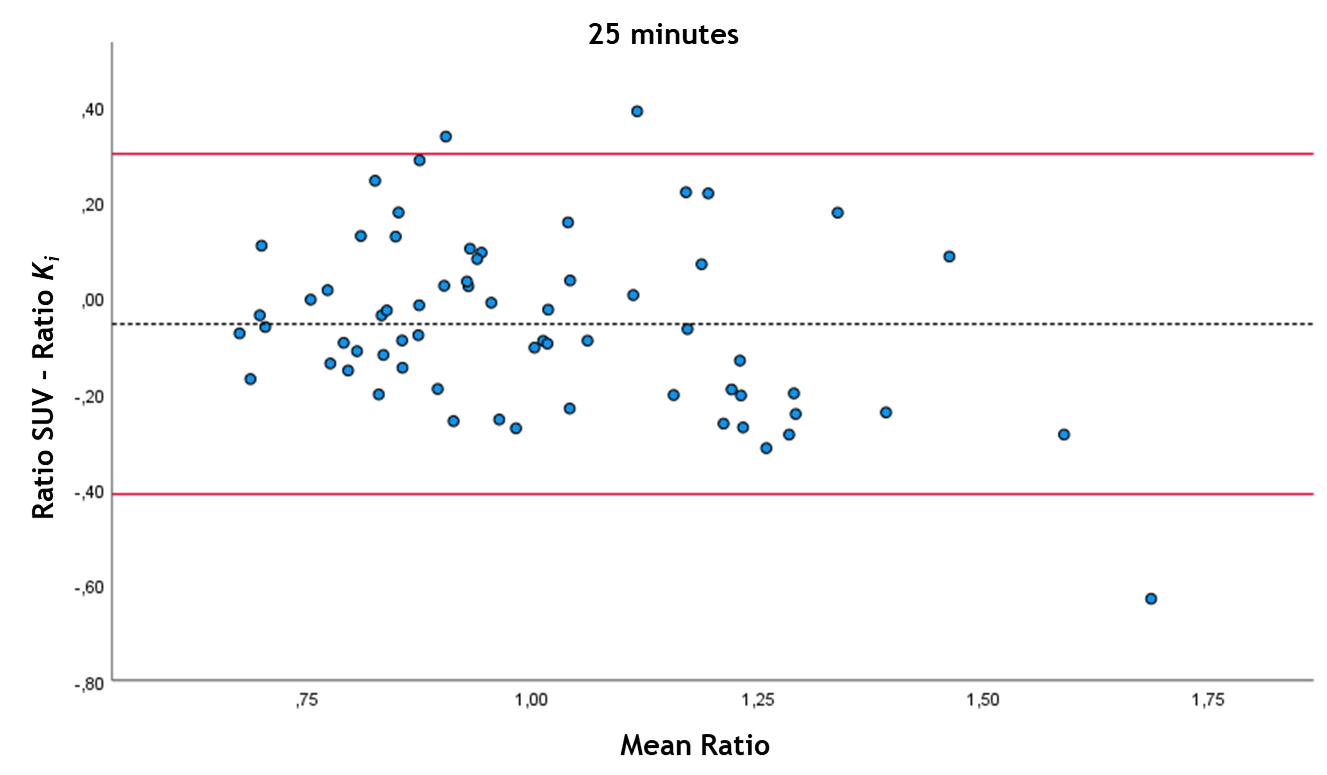
*

***Sup 7.*** *Bland-Altman plot of ratio’s between K_i_ and SUV_peak-LBM_ at 25 minutes after tracer injection.*

*
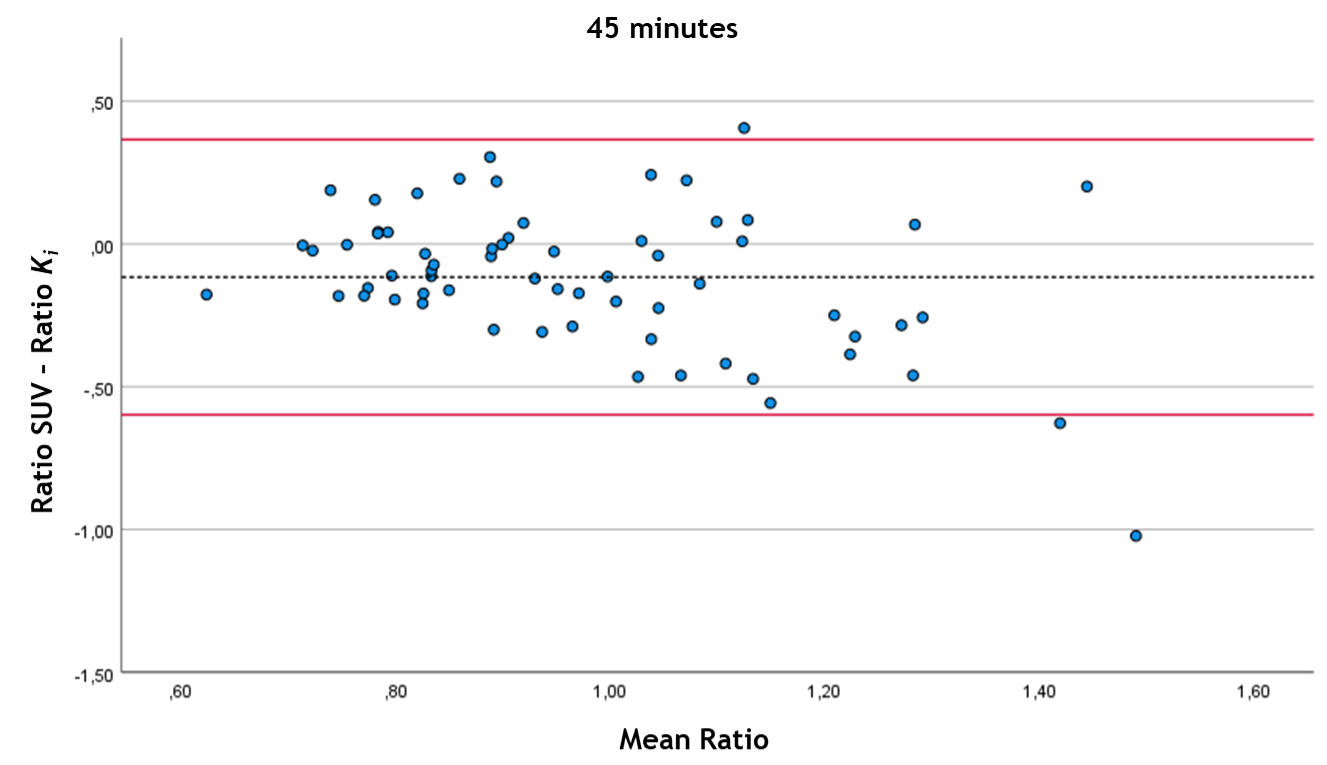
*

***Sup 8.*** *Bland-Altman plot of ratio’s between K_i_ and SUV_peak-LBM_ at 45 minutes after tracer injection*
